# GPR68, A Proton-sensing GPCR, Mediates Acid-induced Visceral Nociception

**DOI:** 10.1016/j.jcmgh.2025.101671

**Published:** 2025-11-04

**Authors:** Luke W. Paine, Rohit Gupta, James P. Higham, Javier Aguilera-Lizarraga, Anne Ritoux, Thomas Pritchard, Samuel Nicholson, James R.F. Hockley, Tim Raine, Martin Hausmann, Kyle Bednar, Gerhard Rogler, Fraser Welsh, Ewan St John Smith, David C. Bulmer

**Affiliations:** 1 Department of Pharmacology, University of Cambridge, Cambridge, United Kingdom; 2 Department of Gastroenterology, Addenbrookes Hospital, Cambridge University Teaching Hospitals, Cambridge, United Kingdom; 3 Department of Gastroenterology and Hepatology, USZ, University of Zurich, Zurich, Switzerland; 4 Respiratory and Immunology, BioPharmaceuticals R&D, AstraZeneca, Gaithersberg, Maryland; 5 Neuroscience, BioPharmaceuticals R&D, AstraZeneca, Cambridge, United Kingdom

**Keywords:** Colitis, Nociceptors, Pain, Visceral Hypersensitivity

## Abstract

**Background & Aims:**

Localised acidification from immune cell infiltration and heightened glycolysis contributes to colitis pathology by activating acid-sensing receptors such as G protein-coupled receptor 68 (GPR68), a proton-sensing G protein-coupled receptor (GPCR) expressed on immune and stromal cells. Single-cell RNA sequencing (RNA-seq) analysis revealed GPR68 is also expressed in colonic sensory neurons, prompting us to investigate its role in acid-induced colonic nociception.

**Methods:**

Expression of GPR68 in colonic nociceptors and tissue from people with colitis was confirmed by in silico analysis of our RNA-seq databases. Its contribution to disease activity was assessed using the acute dextran sulphate sodium (DSS) model of colitis. Acid-evoked sensory signalling was evaluated via colonic afferent recordings and Ca^2+^ imaging in DRG neurons from wild-type and GPR68^-/-^ mice, supported by pharmacological studies using Ogerin (a GPR68 positive allosteric modulator) and Ogremorphin (a GPR68 antagonist).

**Results:**

RNA-seq analysis showed GPR68 is robustly expressed in *Trpv1*^+^ colonic nociceptors and upregulated in tissue from people with inflammatory bowel disease, consistent with reduced disease activity in DSS-treated GPR68^-/-^ mice. Genetic deletion of GPR68 abolished colonic afferent responses to acid, which were also attenuated by Ogremorphin and enhanced by Ogerin. In Ca^2+^-free buffer, dorsal root ganglion neurons from GPR68^-/-^ mice or those pretreated with Ogremorphin showed significantly reduced acid-evoked intracellular Ca^2+^ responses. By contrast, the colonic afferent and dorsal root ganglion Ca^2+^ response (in Ca^2+^-containing buffer) to capsaicin was comparable between tissue from wild-type and GPR68^-/-^ mice highlighting the involvement of divergent proton-dependent cellular signaling cascades.

**Conclusions:**

These findings identify GPR68 as a key mediator of acid-induced colonic nociception and highlight its potential as a therapeutic target for the treatment of pain in colitis.

## Graphical abstract

**Figure:**
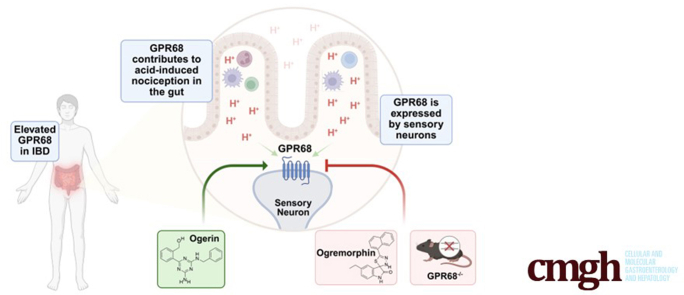

**Summary**

We demonstrate that the proton-sensing receptor G protein-coupled receptor 68 (GPR68) mediates acid-induced signalling in colonic sensory neurons. Genetic and pharmacological modulation confirms GPR68’s role in visceral nociception and supports its potential as a therapeutic target for pain management in colitis.

**What You Need to Know**

**Background**

Local acidification occurs during intestinal inflammation. We investigated whether the proton-sensing G protein-coupled receptor, G protein-coupled receptor 68 (GPR68), expressed by colonic sensory neurons, contributes to acid-induced nociception.

**Impact**

Using complementary genetic and pharmacological approaches, we demonstrate that GPR68 mediates acid-evoked activation of sensory neurons and colonic afferents, highlighting its potential as a therapeutic target for novel visceral analgesics.

**Future Directions**

GPR68 antagonists may alleviate colitis-associated pain. Future studies should elucidate the mechanistic pathways linking GPR68 to nociception and evaluate its translational potential in models of visceral pain.

Inflammatory bowel disease (IBD) is characterized by chronic inflammation of the gastrointestinal (GI) tract and is accompanied by acidification of the gut lumen and mucosa^1^, ^2^, ^3^, ^4^ with studies reporting colonic pH values ranging from pH 6.1 to 7.5 in healthy controls, 4.8 to 7.3 in ulcerative colitis (UC), and 5.3 to 6.5 in Crohn’s disease (CD),^1^^,^^2^ consistent with elevated fecal lactate levels^5^ and reduced fecal pH.^6^ Local tissue acidosis is linked to IBD pathogenesis and can be attributed to the increased glycolytic activity, alongside reduced tissue perfusion and hypoxia,^7^ which occurs in response to the influx of immune cells, tissue inflammation and edema.^8^ Furthermore, acidity drives pro-inflammatory gene expression in infiltrating immune cells through multiple acid-sensing mechanisms,^9^^,^^10^ with emerging evidence suggesting that the upregulation and increased activity of proton-sensing G protein-coupled receptors (GPCRs), such as G protein-coupled receptor 68 (GPR68), contribute to these changes in immune cell behaviour and ultimately the pathogenesis of IBD.^7^

The orphan proton-sensing GPCRs, GPR4, GPR65 (TDAG8), GPR68 (OGR1) and GPR132 (G2A), all have recognized roles in colitis pathophysiology^7^^,^^11^ and other inflammatory and neuropathic pain conditions.^12^, ^13^, ^14^, ^15^ Although often classified with these receptors, GPR132 differs functionally and structurally, showing weak proton sensitivity and being primarily activated by oxidized fatty acids.^16^ Upon extracellular acidification, protonation of histidine residues in the extracellular domain of GPR4, GPR65, and GPR68 initiates receptor activation, a conserved feature among these core proton sensors.^17^ Recent computational and in vitro studies have expanded this model, implicating multiple cooperative interactions between histidine and acidic residues, across the extracellular and transmembrane domains, as critical for proton-sensing and conformational activation.^18^, ^19^, ^20^

Intracellularly, GPR4 and GPR65 primarily couple to Gα_s_ proteins, whereas GPR68 preferentially couples to Gα_q/11_ proteins triggering phospholipase C (PLC) activation, inositol triphosphate (IP_3_) production, and subsequent Ca^2+^ release from intracellular stores, which can lead to pro-inflammatory signaling cascades.^17^ Consistent with this, GPR68 activation perpetuates intestinal inflammation, and its genetic deletion confers resistance to fibrosis^21^ and gut inflammation in IL-10^-/-^ spontaneous colitis models.^22^ Additionally, pharmacological inhibition of GPR68 decreases the severity of both acute and chronic dextran sulphate sodium (DSS)-induced colitis.^23^ GPR68 signalling also drives pro-inflammatory gene expression in immune and stromal cells, amplifying gut inflammation, and its expression is upregulated in colonic tissue during hypoxia.^24^, ^25^, ^26^ Consequently, GPR68 has emerged as a promising therapeutic target for inflammation in IBD.

In keeping with the underlying inflammation and tissue damage, abdominal pain is a leading cause of morbidity in colitis, and its treatment represents a significant unmet clinical need facilitated by the contraindicated use of opioid and nonsteroidal anti-inflammatory drug (NSAID)-based pain killers for use in people with IBD. Consequently, there is an increasing call from patient advocacy groups for the development of treatment strategies that can simultaneously address both inflammation and pain in IBD.

Given that acid-sensing is a conserved function of pain sensing neurons across species and a defining feature of polymodal nociceptors, facilitated by their expression of acid-sensitive ion channels and GPCRs,^27^ we sought to understand whether proton-sensing GPCRs such as GPR68 might also be responsible for the activation of colonic nociceptors by acid, and as such contribute to the activation of both nerve endings and immune cells following acidification of the bowel in individuals with colitis. GPR68 has previously been implicated in chemotherapy-induced neuropathy,^28^ and its heightened expression in sensory neurons^29^ and GI tissue^22^ compared with other proton-sensing GPCRs, supports a possible pro-nociceptive role for GPR68. Here, using complementary genetic and pharmacological approaches, we assess the contribution of GPR68 to acid-evoked sensory neuron and colonic afferent activation, highlighting its potential as a therapeutic target for the development of novel visceral analgesics.

## Results

### In Silico and In Vivo Analyses Implicate GPR68 in IBD Pathology and Reveal Coexpression With Trpv1 in Murine Colon-projecting Sensory Neurons

**Figure 1:**
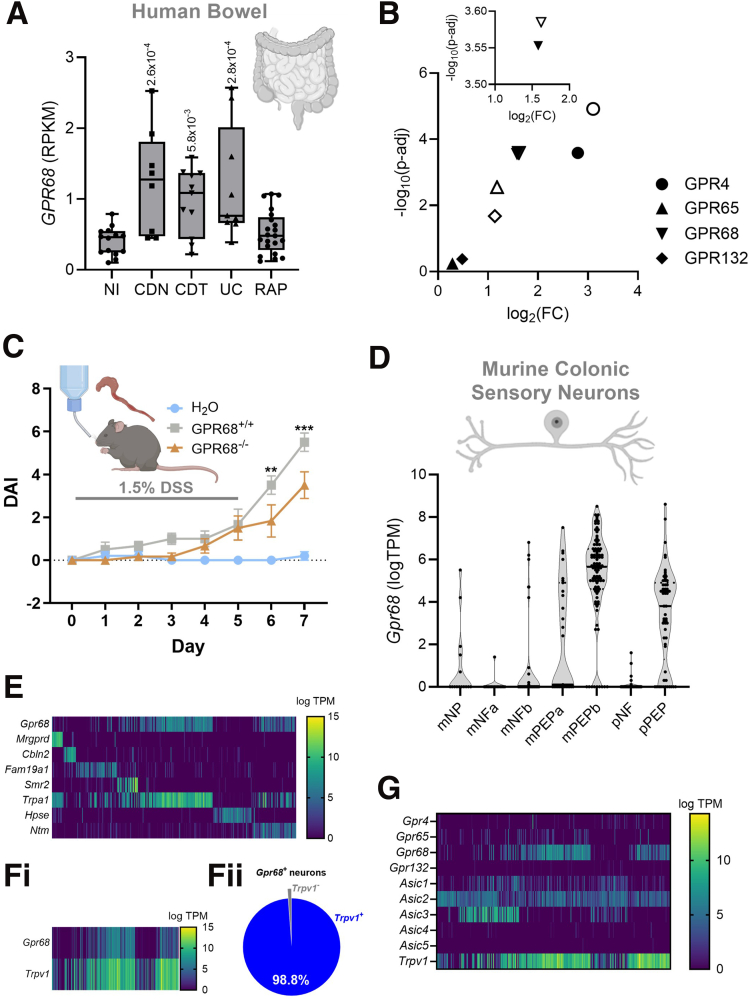
**GPR68 is upregulated in bowel tissue from individuals with colitis and enriched in murine colon-innervating sensory neurons.** (*A*) Expression (RPKM, reads per kilobase million) of *GPR68* in colonic biopsies taken from people with NI, UC, CDN, CDT, and RAP. Median, interquartile range (*box*), and full range (*whiskers*) is shown. (*GPR68* CDN vs NI; *P* = 2.6 × 10^-4^; CDT vs NI; *P* = 5.8 × 10^-3^; UC vs NI; *P* = 2.8 × 10^-4^; 1-way ANOVA (main effect: *P* = 6.53 × 10^-5^; F(4, 58) = 3.579) with Benjamini-Hochberg post-hoc test). Data redrawn from Higham et al.^30^ (*B*) Expression of selected proton-sensing GPCRs in UC (*filled symbols*) and CDN (*open symbols*) given as log2 fold change compared with noninflamed controls with the associated FDR-adjusted *P*-value (as −log10). *Inset*: expanded view highlighting GPR68 expression.^30^ (*C*) DAI over the course of 1.5%-induced colitis in GPR68^+/+^ and GPR68^-/-^ mice (GPR68^+/+^ vs GPR68^-/-^ Day 6; *P* = .003∗∗; Day 7; *P* = .0003∗∗∗; 2-way ANOVA (main effect: *P* ≤ .0001; F(2, 112) = 40.57) with Tukey’s post-hoc test). The H_2_O control group consisted of GPR68^+/+^ mice only. (*D*) Violin plot showing the expression (logTPM) of *Gpr68* in the murine colonic afferent populations identified by Hockley et al.^31^ (*E*) Heatmap showing the expression (logTPM) of *Gpr68* and markers of each of the murine colonic afferent populations. Heatmap (*Fi*) and Chart (*Fii*) showing the fraction of *Gpr68*-expressing sensory neurons which co-express *Trpv1*. Data redrawn from Hockley et al.^31^ (*G*) Heatmap showing the expression (logTPM) of *Gpr4*, *Gpr65*, *Gpr68*, and *Gpr132*, as well as *ASIC1-5* and *Trpv1* in murine colonic sensory neurons (data redrawn from Hockley et al^31^).

In silico analysis of transcript expression in colonic biopsies revealed a 3-fold increase in *GPR68* mRNA in samples from individuals with UC compared with noninflamed controls (NI) (*P* = 2.8 × 10^-4^, 1-way analysis of variance [ANOVA] with Benjamini-Hochberg post-hoc test) (Figure 1*A*). *GPR68* expression was also elevated in both drug-naïve and drug-treated CD samples (CDN and CDT, respectively) (NI vs CDN: *P* = 2.6 × 10^-4^; NI vs CDT: *P* = 5.8 × 10^-3^; 1-way ANOVA with Benjamini-Hochberg post-hoc test; data redrawn from Higham et al^30^) (Figure 1*A*). This increase in GPR68 expression in colitic tissue surpassed that of the proton-sensing GPCRs, GPR132 and GPR65 (Figure 1*B*).

**Figure 2:**
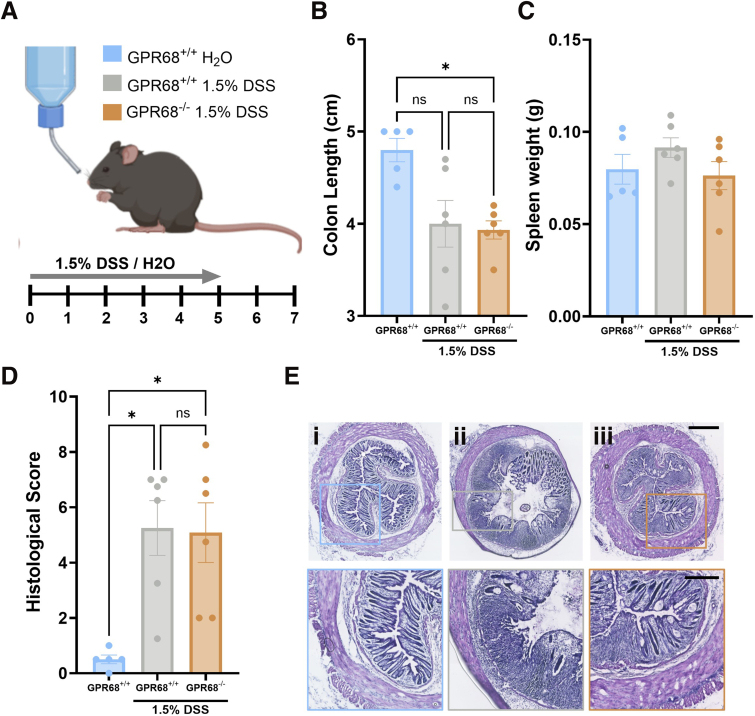
**Additional parameters of the DSS-induced colitis model in GPR68^+/+^ and GPR68^-/-^ mice.** (*A*) Schematic representation of the DSS model and experimental timeline. (*B*) Colon length and spleen weight (*C*) measured on day 7 following administration of H_2_O or 1.5% DSS in GPR68^+/+^ and GPR68^-/-^ mice (colon length: H_2_O control vs GPR68^+/+^ DSS: *P* = .0760; H_2_O control vs GPR68^-/-^ DSS: *P* = .0207; GPR68^+/+^ DSS vs GPR68^-/-^ DSS: *P* ≥ .9999; Kruskal-Wallis test (main effect: *P* = .01; H = 8.129) with Dunn’s multiple comparison (Z = 2.236, 2.702, 0.4886, respectively) (spleen weight: H_2_O control vs GPR68^+/+^ DSS: *P* = .6834; H_2_O control vs GPR68^-/-^ DSS: *P* ≥ .9999; GPR68^+/+^ DSS vs GPR68^-/-^ DSS: *P* = .4333; Kruskal-Wallis test (main effect: *P* = .3045; H = 2.467) with Dunn’s multiple comparison (Z = 1.206, 0.1855, 0.1.460, respectively). (*D*) Histological scores of colon sections based on inflammation, crypt damage, and ulceration (H_2_O control vs GPR68^+/+^ DSS: *P* = .0156; H_2_O control vs GPR68^-/-^ DSS: *P* = .0156; GPR68^+/+^ DSS vs GPR68^-/-^ DSS: *P* ≥ .9999; Kruskal-Wallis test (main effect: *P* = .0019; H = 10.11) with Dunn’s multiple comparison (Z = 2.795, 2.795, 0.000, respectively). (*G*) Representative H&E staining of colon sections across experimental groups (*i*) H_2_O control; (*ii*) GPR68^+/+^ 1.5% DSS; and (*iii*) GPR68^-/-^ 1.5% DSS. Scale bars: full cross-sections = 500 μm, higher magnification insets = 250 μm. (N = 5–6 animals).

To investigate the functional role of GPR68 in colitis, we used a 1.5% DSS-induced colitis model.^31^ GPR68^-/-^ mice exhibited significantly reduced disease activity compared with wild-type controls on days 6 and 7 (GPR68^+/+^ vs GPR68^-/-^ Day 6, *P* = .003; Day 7, *P* = .0003; 2-way ANOVA with Tukey’s post-hoc test; N = 6) (Figure 1*C*). No significant differences were observed in colon length, spleen weight, or histological inflammation scores (Figure 2).

Further supporting a role for GPR68 in colitis-associated nociception, we found that *Gpr68* expression was enriched in peptidergic subpopulations of colonic sensory neurons (Figure 1*D–E*) and showed high co-expression with *Trpv1*, a marker of nociceptive afferents (Figure 1*F*).

Comparison of GPR68 with other proton-sensing GPCRs (GPR4, GPR65, and GPR132) revealed that, although expression of each receptor was upregulated to varying degrees in colitic tissue, GPR68 was the most highly expressed proton-sensing GPCR in colonic sensory neurons, and shows similarly high expression to certain other acid sensors, highlighting its potential contribution to both inflammation and visceral pain (Figure 1*B, G*).

### GPR68 Contributes to Acid-evoked Intracellular Ca^2+^ Responses in Dorsal Root Ganglion Sensory Neurons

**Figure 3:**
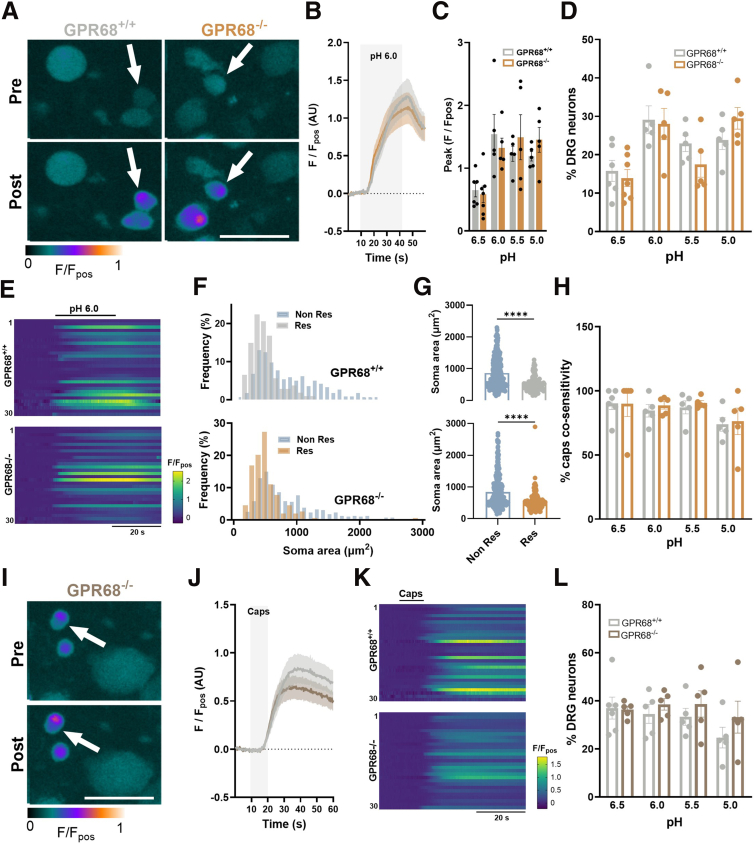
**Characterisation of acid- and capsaicin- evoked [Ca^2+^]_i_ mobilization in DRG neurons isolated from GPR68^+/+^ and GPR68^-/-^ mice.** (*A*) False-colored images showing Fluo-4 fluorescence during the application of normal pH 6.0 ECS to DRG neurons isolated from GPR68^+/+^ and GPR68^-/-^ mice. *White arrows* highlight cells responding to pH 6.0. Scale bar: 50 μM. (*B*) Averaged traces of pH 6.0 response and (*C*) peak responses across the pH range in DRG neurons isolated from GPR68^+/+^ and GPR68^-/-^ mice (GPR68^+/+^ vs GPR68^-/-^*P* = .9984, .9117, .8385, .8443 [left to right]; 1-way ANOVA [main effect: *P* = .0031, F(7, 35) = 3.901] with Sidak’s multiple comparisons test; n = 5–7 dishes from N = 5 animals). (*D*) The proportion of GPR68^+/+^ and GPR68^-/-^ DRG neurons responding to pH 6.5–5.0. (GPR68^+/+^ vs GPR68^-/-^*P* = .9808, .9984, .6238, .5866 [left to right]; 1-way ANOVA [main effect: *P* = .0008, F(7, 35) = 4.701] with Sidak’s multiple comparisons test; n = 5–7 dishes from N = 5 animals). (*E*) Heatmap showing Fluo-4 fluorescence during the application of pH 6.0 for 30 randomly selected DRG neurons for 30 seconds isolated from GPR68^+/+^ (*top*) and GPR68^-/-^ (*bottom*) mice. (*F*) Cell size frequency distribution and (*G*) scatter dot plot of average soma areas (μm^2^) of responders and nonresponders to pH 6.0 in both genotypes (GPR68^+/+^*P* ≤ .0001 [U = 10075]; GPR68^-/-^*P* ≤ .0001 [U = 17675]; Mann-Whitney Test; n = 445 and n = 548 cells, respectively). (*H*) The proportion of pH 6.0-responding DRG neurons also co-sensitive to capsaicin (GPR68^+/+^ vs GPR68^-/-^*P* ≥ .9999, .9823, .9923, .9983 [left to right]; 1-way ANOVA [main effect: *P* = .3866, F(7, 33) = 1.099] with Sidak’s multiple comparisons test; N = 5–6). (*I*) False-coloured images showing Fluo-4 fluorescence in GPR68^-/-^ DRG neurons in response to capsaicin (1 μM). (*J*) Averaged traces and heatmaps of 30 selected GPR68^+/+^ and GPR68^-/-^ DRG neurons (*K*) portraying responses to capsaicin. (*L*) The proportion of GPR68^+/+^ and GPR68^-/-^ DRG neurons responding to capsaicin (GPR68^+/+^ vs GPR68^-/-^*P* = .9520, .8642, .5481, > .9999 [left to right]; 1-way ANOVA [main effect: *P* = .4100, F(7, 33) = 1.061] with Sidak’s multiple comparisons test; n = 5–6 dishes from N = 5 animals).

Given the expression of *Gpr68* in mouse colonic sensory neurons, we next sought to investigate the contribution of GPR68 to acid-induced intracellular Ca^2+^ mobilization ([Ca^2+^]_i_) in cultured thoracolumbar dorsal root ganglion (DRG) neurons. We first characterized responses to acidic pH and capsaicin in neurons isolated from GPR68^+/+^ and GPR68^-/-^ mice under normal extracellular Ca^2+^ conditions, observing no significant difference in either the peak response or the proportion of DRG neurons responding across the pH range (pH 6.5–5.0) (Figure 3*A–E*) and to capsaicin (Figure 3*I–L*). In both genotypes, acid-sensitive neurons were significantly smaller than nonresponders (*P* ≤ .0001, Mann Whitney tests) (Figure 3*F–G*) and exhibited high capsaicin co-sensitivity (pH 6.0: 84.5% ± 4.9% and 88.8% ± 3.0% for GPR68^+/+^ and GPR68^-/-^, respectively), consistent with the activation of *Trpv1*-positive sensory neurons (Figure 3*A–E*).

**Figure 4:**
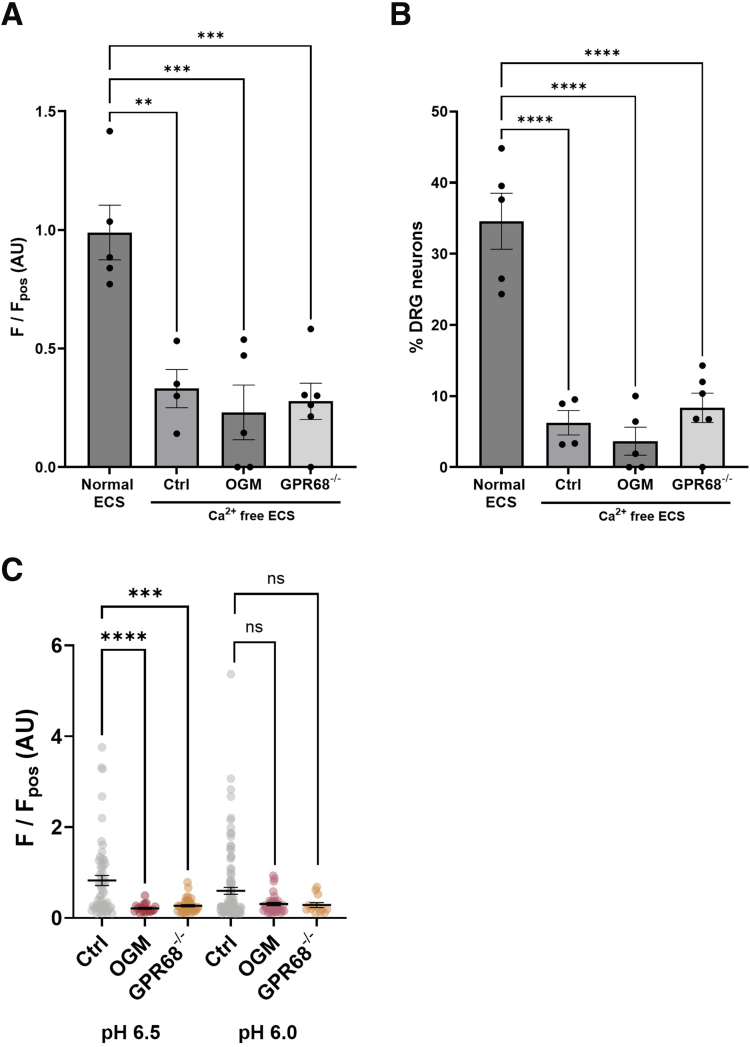
**Attenuation of capsaicin response in DRG neurons following extracellular Ca^2+^ removal.** (*A*) Peak responses and (*B*) the proportion of DRG neurons responding to capsaicin (1 μM) in normal ECS (data taken from Figure 5) and Ca^2+^-free ECS across treatment groups (Peak: normal ECS vs Ca^2+^-free ECS, control [*P* = .0012], vs OGM [*P* = .0002], vs GPR68^-/-^ [*P* = .0002], 1-way ANOVA [main effect: *P* = .0001, F(3, 16) = 13.11] with Dunnett’s multiple comparisons test; n = 4–6 dishes from N = 3–5 animals) (Proportion of DRG neurons: normal ECS vs Ca^2+^-free ECS, control [*P* ≤ .0001], vs OGM [*P* ≤ .0001], vs GPR68^-/-^ [*P* ≤ .0001], 1-way ANOVA [main effect: *P* ≤ .0001, F(3, 16) = 29.79] with Dunnett’s multiple comparisons test; n = 4–6 dishes from N = 3–5 animals). (*C*) Peak responses of individual DRG neurons responding to pH 6.5 and pH 6.0 in control, OGM-treated, and GPR68^-/-^ mice. (∗∗∗*P* < .001, ∗∗∗∗*P* < .0001, Kruskal-Wallis tests with Dunn’s multiple comparisons test).

**Figure 5:**
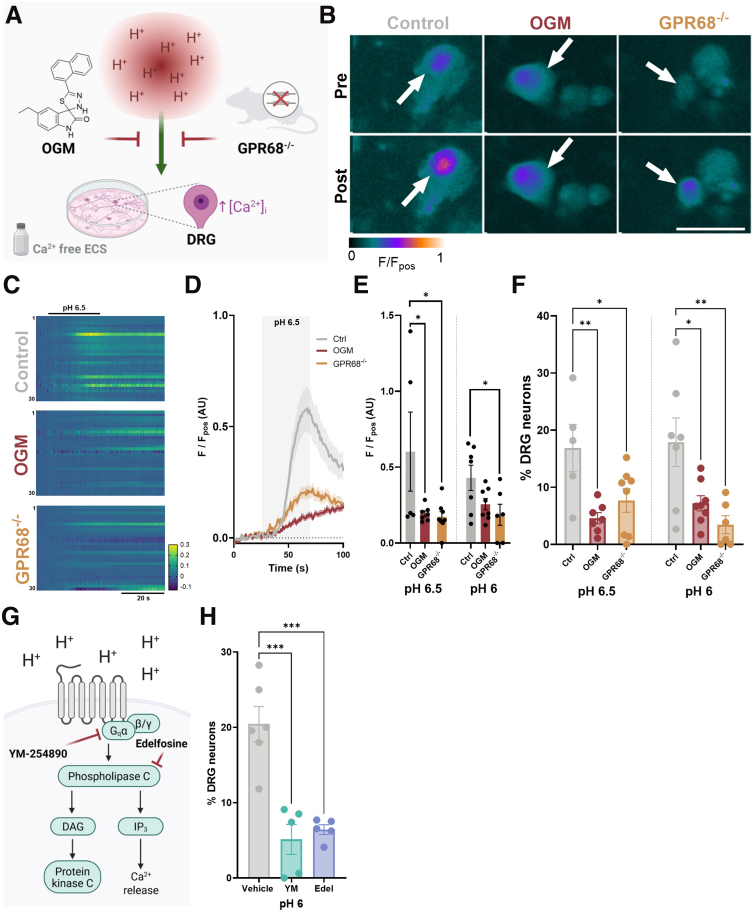
**GPR68 contributes to acid-evoked [Ca^2+^]_i_ increase in DRG sensory neurons following removal of external Ca^2+^.** (*A*) Schematic of the experimental paradigm assessing DRG neuron responses to acidic pH in Ca^2+^-free ECS. (*B*) False-colored images and (*C*) heatmaps (30 randomly selected neurons) showing Fluo-4 fluorescence during the application of Ca^2+^-free ECS adjusted to pH 6.5 in GPR68^+/+^ and GPR68^-/-^ DRG neurons and following pretreatment with GPR68 antagonist OGM. *White arrows* highlight cells responding to pH 6.5. Scale bar: 50 μM. (*D*) Averaged response profiles and peak responses (*E*) to acidic pH across treatment conditions (pH 6.5 control vs OGM: *P* = .0499; Control vs GPR68^-/-^: *P* = .0340; 1-way ANOVA [main effect: *P* = .0403; F(2, 17) = 3.903] with Dunnett’s multiple comparisons test. pH 6.0 control vs OGM: *P* = .1101; control vs GPR68^-/-^: *P* = .0334; 1-way ANOVA [main effect: *P* = .0451; F(2, 18) = 3.7] with Dunnett’s multiple comparisons test; n = 5–8 dishes from N = 5–6 animals) (*F*) The proportion of GPR68^+/+^ and GPR68^-/-^ DRG neurons responding to pH 6.5 and pH 6.0 and following pre-treatment with OGM (pH 6.5 control vs OGM: *P* = .0051; control vs GPR68^-/-^: *P* = .0274; 1-way ANOVA [main effect: *P* = .0085; F(2, 17) = 6.392] with Dunnett’s multiple comparisons test. pH 6.0 control vs OGM: *P* = .0189; control vs GPR68^-/-^: *P* = .0038; 1-way ANOVA [main effect: *P* = .0048; F(2, 18) = 7.281] with Dunnett’s multiple comparisons test; n = 5–8 dishes from N = 5–6 animals). (*G*) Schematic and (*H*) proportion of DRG neurons responding to pH 6.0 in the presence of vehicle, YM-254890 (YM; 100 nM) or edelfosine (Edel; 10 μM) (vehicle vs YM: *P* = .0001; vehicle vs Edel: *P* = .0003; 1-way ANOVA [main effect: *P* ≤ .0001; F(2, 13) = 21.05] with Dunnett’s multiple comparisons test; n = 5–6 dishes from N = 4–5 animals).

Although no differences in pH responses were observed under normal extracellular Ca^2+^ conditions, repeating experiments in Ca^2+^ free buffer, thereby restricting [Ca^2+^]_i_ mobilization to intracellular stores (confirmed by loss of response to capsaicin) (Figure 4), revealed a significant decrease in the proportion of DRG neurons responding to pH 6.5 and pH 6.0 in neurons isolated from GPR68^-/-^ mice compared with wild-type mice or following pretreatment with the GPR68 antagonist, Ogremorphin in wild-type mice (OGM; pH 6.5: control vs OGM: *P* = .0051; Control vs GPR68^-/-^: *P* = .0274; 1-way ANOVA with Dunnett’s multiple comparisons test; N = 5–8) (Figure 5*A–F*). Additionally, the magnitude of the response in the remaining acid-sensitive neurons was also significantly reduced in cells from GPR68^-/-^ mice or OGM-treated cells (pH 6.5: control vs OGM: *P* = .0499; control vs GPR68^-/-^: *P* = .0340; 1-way ANOVA with Dunnett’s multiple comparisons test; N = 5–8) (Figure 5*A–F*). These results confirm that GPR68 contributes to acid-induced [Ca^2+^]_i_ mobilization in DRG sensory neurons.

Additionally, these findings support a G_q_-coupled mechanism of intracellular Ca^2+^ release (Figure 5*G*), as inhibition of G_q_ with YM-254890 significantly reduced the proportion of DRG neurons exhibiting pH 6-induced Ca^2+^ transients (*P* = .0001; 1-way ANOVA with Dunnett’s multiple comparisons test; N = 4–5) (Figure 5*H*), complemented by blockade of PLC with edelfosine evoking a similar attenuation (*P* = .0003; 1-way ANOVA with Dunnett’s multiple comparisons test; N = 4–5) (Figure 5*H*). Experiments were performed using pH 6.5 and pH 6.0 as these concentrations of protons have previously been reported to elicit robust GPR68 activity^17^ with minimal transient receptor potential vanilloid 1(TRPV1) activity.^32^

### Colonic Afferent Responses to Acidic pH Are Attenuated in GPR68^-/-^ Mice and Inhibited in Wild-type Mice by GPR68 Inhibition

Having established the presence of GPR68 mediated acid-evoked stimulation of DRG sensory neurons, we next investigated the contribution of GPR68 to acid-evoked stimulation of colonic afferents using the lumbar splanchnic nerve (LSN) preparation.

**Figure 6:**
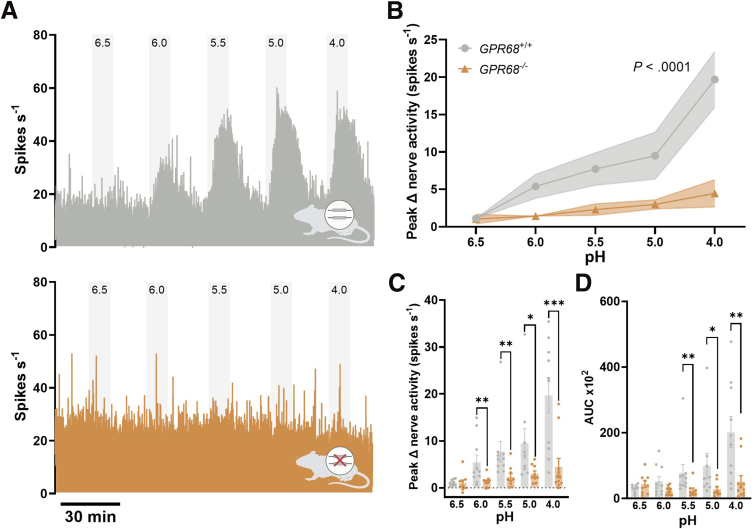
**Colonic afferents from GPR68^-/-^ mice exhibit reduced acid-sensitivity.** (*A*) Representative rate histograms and (*B*) mean response profiles of GPR68^+/+^ (*gray*) and GPR68^-/-^ (*orange*) colonic afferent activity in response to bath application of pH 6.5–4.0; shaded area is SEM (GPR68^+/+^ vs GPR68^-/-^ main effect: *P* ≤ .0001; 2-way ANOVA; F(1, 87) = 26.47). (*C*) Peak afferent activity at each pH (GPR68^+/+^ vs GPR68^-/-^ unpaired *t*-test [or nonparametric equivalent]; pH 6.5, *P* = .9126, t, df = .1115, 16; pH 6.0, *P* = .0074 (U = 18); pH 5.5, *P* = .003 (U = 10); pH 5.0, *P* = .0106 (U = 12); pH 4.0, *P* = .0008 (U = 10); N = 9–11 animals). (*D*) AUC for afferent responses across the pH range (GPR68^+/+^ vs GPR68^-/-^ unpaired *t*-test (or nonparametric equivalent); pH 6.5, *P* = .2430, t, df = 1.212, 16; pH 6.0, *P* = .1145 (U = 32); pH 5.5, *P* = .003 (U = 10); pH 5.0, *P* = .0106 (U = 12); pH 4.0, *P* = .0015 (U = 12); N = 9–11 animals). U refers to the Mann-Whitney *U* statistic.

Repeat application of acidic Krebs buffer (pH 6.5–4.0) elicited a robust increase in colonic afferent activity in tissue from GPR68^+/+^ mice, which was absent in tissue from GPR68^-/-^ mice (*P* ≤ .0001; 2-way ANOVA; N = 9–11) (Figure 6*A–B*). This reduction was reflected in both the peak change in nerve activity and area under the curve (AUC) of the colonic afferent response (peak activity: pH 6.0: 1.277 ± 0.339 spikes/s; *P* = .0074; pH 4.0: 4.452 ± 1.82 spikes/s; *P* = .0008; AUC: pH 5.5; *P* = .003; Mann-Whitney tests; N = 9–11) (Figure 6*C–D*).

**Figure 7:**
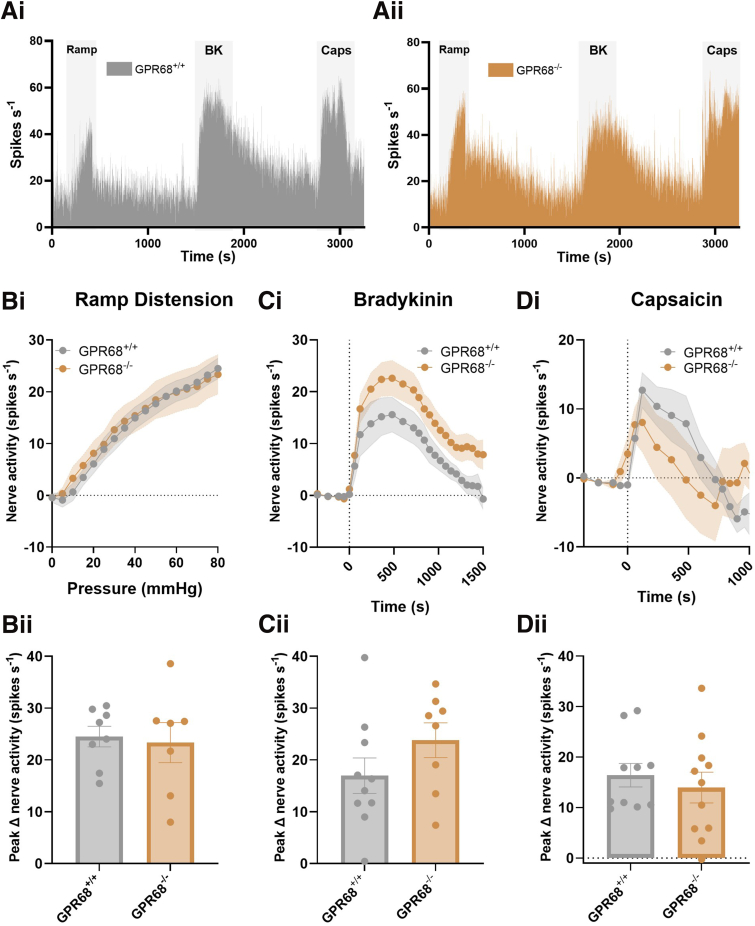
**GPR68^+/+^ and GPR68^-/-^ colonic afferent activity is comparable in response to mechanical and chemical (bradykinin and capsaicin) stimuli.** (*A*) Representative rate histogram of (*i*) GPR68^+/+^ and (*ii*) GPR68^-/-^ colonic afferent responses to ramp distension (Ramp), bradykinin (BK; 1 μM), and capsaicin (Caps; 1 μM). (*Bi*) Response profile (*P* = .8246, 2-way ANOVA; F(1, 13) = .05113; N = 7–8) and (*Bii*) peak change in afferent activity at 80 mmHg in response to ramp distension (*P* = .7861 (t, df = .2770, 13), unpaired *t*-test; N = 7–8). (*Ci*) Response profile (*P* = .0947, 2-way ANOVA; F(1, 15) = 3.182; N = 8–10) and (*Cii*) peak change in afferent activity in response to bradykinin stimulation (*P* = .1785 (t, df = 1.407, 16), unpaired *t*-test; N = 8–10). (*Di*) Response profile (*P* = .7187, 2-way ANOVA; F(1, 19) = .1337; N = 10–11) and (*Dii*) peak change in nerve activity in response to capsaicin (1 μM) stimulation (*P* = .5116, Mann-Whitney test (U = 45); N = 10–11).

**Figure 8:**
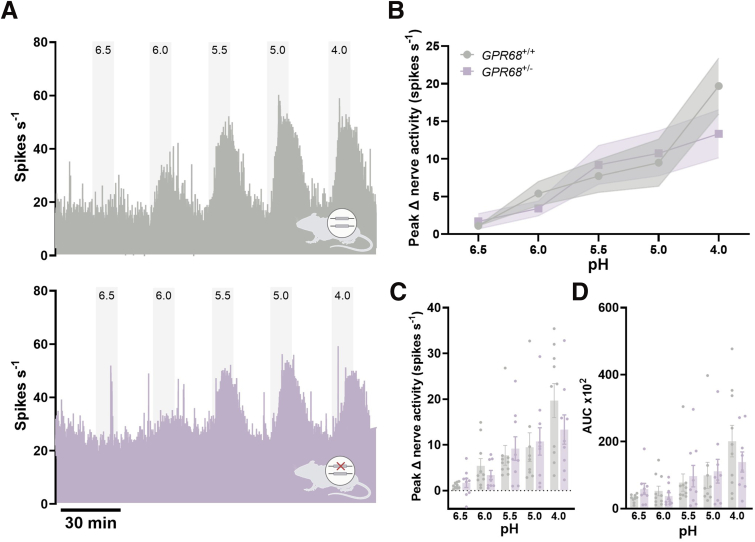
**Comparable colonic afferent responses to acid in GPR68^+/+^ and GPR68^+/-^ mice.** (*A*) Representative rate histograms and (*B*) mean response profiles of GPR68^+/+^ (*gray*) and GPR68^+/-^ (*lilac*) colonic afferent activity in response to pH 6.5-4.0; shaded area is SEM. (GPR68^+/+^ vs GPR68^+/-^ main effect: *P* = .5185; 2-way ANOVA; F(1, 83) = .4204). (*C*) Peak afferent activity at each pH (GPR68^+/+^ vs GPR68^+/-^ unpaired *t*-test [or nonparametric equivalent]; pH 6.5, *P* = .5770, t, df = .5694, 16; pH 6.0, *P* = .2428 (U = 30); pH 5.5, *P* = .7197 (U = 40); pH 5.0, *P* = .6665 (U = 35); pH 4.0, *P* = .2179, t, df = 1.279, 17; N = 9–10 animals). (*D*) AUC for responses across the pH range (GPR68^+/+^ vs GPR68^+/-^ unpaired *t*-test [or nonparametric equivalent]; pH 6.5, *P* = .0939 (U = 21); pH 6.0, *P* = .4470 (U = 35); pH 5.5, *P* = .9682 (U = 44); pH 5.0, *P* = .8633 (U = 38); pH 4.0, *P* = .4002 (U = 34); N = 9–10 animals). Wild-type data shown here are the same as those presented in Figure 6 and are included for direct comparison with GPR68^+/-^ mice.

Importantly no significant differences were seen in the LSN response to ramp distension (*P* = .7861; unpaired *t*-test; N = 7–8), bradykinin (*P* = .1785; unpaired *t*-test; N = 8–10), or capsaicin (*P* = .5116; Mann-Whitney test; N = 10–11) between GPR68^+/+^ and GPR68^-/-^ preparations, confirming that the loss of acid-evoked colonic afferent activity was stimulus-specific and indicating that responses to noxious stimuli and GPCR signalling in general remained intact (Figure 7). Furthermore, acid-evoked responses in tissue from GPR68^+/-^ mice were comparable to those in wild-type controls (*P* = .5185; 2-way ANOVA; N = 9–10) (Figure 8).

**Figure 9:**
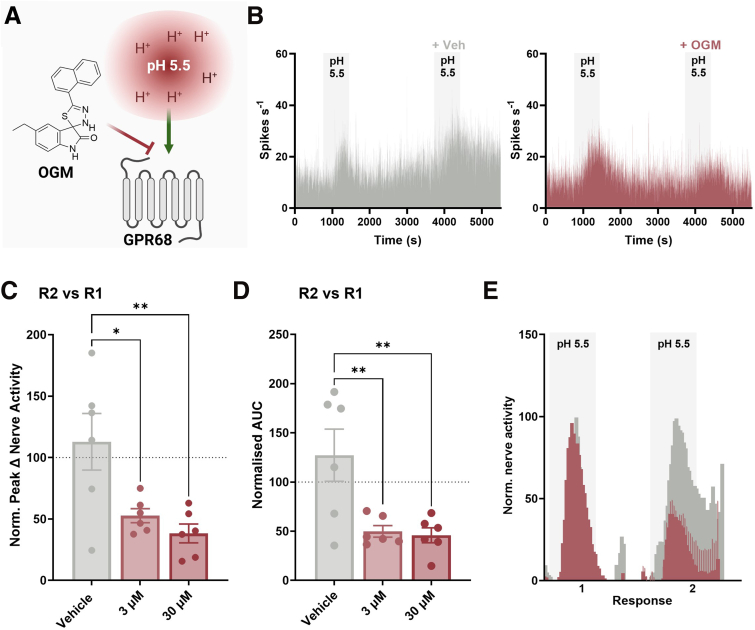
**GPR68 antagonist, OGM, attenuates colonic afferent responses to acid.** (*A*) Schematic of the experimental paradigm assessing colonic afferent responses to bath application of pH 5.5 in the presence and absence of the GPR68 antagonist, OGM. (*B*) Representative rate histograms of repeated stimulation with pH 5.5. The second response consisted of pretreatment with vehicle control (DMSO) (*bottom*) or OGM (*top*). (*C*) Peak change in afferent activity and (*D*) AUC of response 2 (in the presence of OGM or vehicle) expressed as a percentage of the peak response 1 (pH 5.5 alone) (Peak change in afferent activity: vehicle vs 3 μM OGM, *P* = .0183; vehicle vs 30 μM OGM, *P* = .0044; 1-way ANOVA [main effect: *P* = .0054; F(2, 15) = 7.537] with Dunnett’s multiple comparison test) (AUC: vehicle vs 3 μM OGM, *P* = .0081; vehicle vs 30 μM OGM, *P* = .0057; 1-way ANOVA [main effect: *P* = .0045; F(2, 15) = 7.928] with Dunnett’s multiple comparison test). (*E*) Averaged response profiles to pH 5.5 before (response 1) and during (response 2) treatment with vehicle (*gray*) or 3 μM OGM (*red*). (N = 6 animals per group).

Complementary to the data obtained using transgenic knockout mice, pharmacological blockade of GPR68 with the selective GPR68 antagonist, OGM (Figure 9*A–B*) significantly attenuated the peak afferent response (vehicle vs 3 μM OGM; *P* = .0183; vehicle vs 30 μM OGM; *P* = .0044; 1-way ANOVA with Dunnett’s multiple comparisons test; N = 6) (Figure 9*C*) and area under the curve for afferent activity compared with vehicle pretreatment following application of pH 5.5 acidified buffer (vehicle vs 3 μM OGM; *P* = .0081; vehicle vs 30 μM OGM; *P* = .0057; 1-way ANOVA with Dunnett’s multiple comparisons test; N = 6) (Figure 9*D–E*).

Notably, repeat applications of pH 5.5 evoked consistent responses in control LSN recordings, with no significant difference observed across the 3 applications, confirming the reproducibility of the stimulus (comparison not shown).

**Figure 10:**
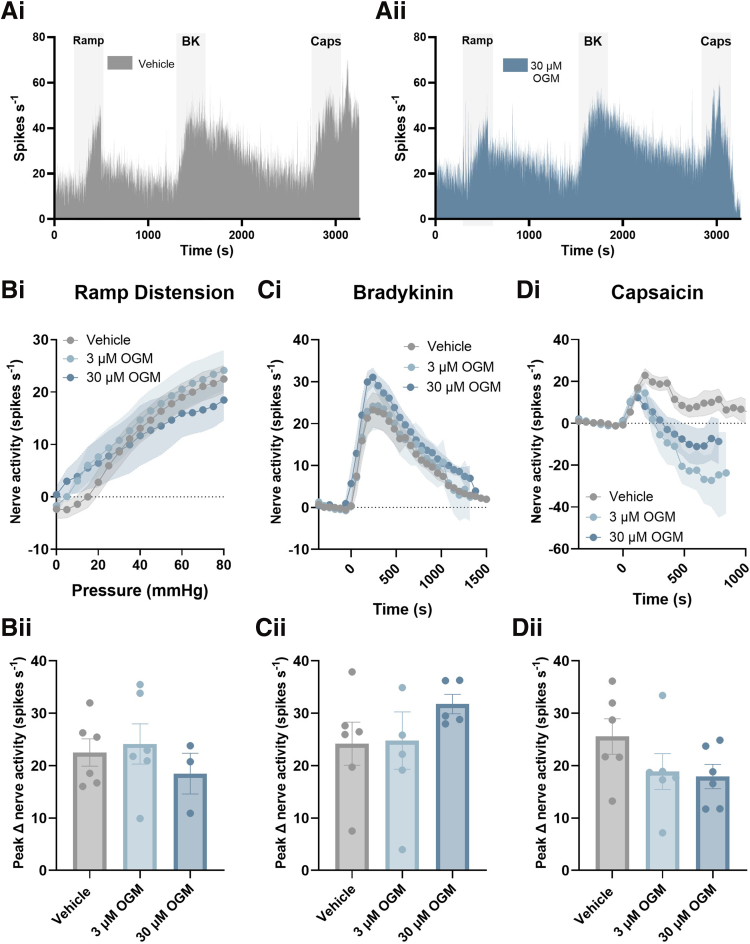
**Comparable colonic afferent responses to mechanical and chemical (bradykinin and capsaicin) stimuli following treatment with OGM.** (*A*) Representative rate histogram of colonic afferent responses to ramp distension (Ramp), bradykinin (BK; 1 μM), and capsaicin (Caps; 1 μM) following treatment with 3 μM or 30 μM OGM (GPR68 antagonist) or vehicle. (*Bi*) Afferent response profile (*P* = .7647; 2-way ANOVA; N = 3–6) and (*Bii*) peak change in afferent activity at 80 mmHg in response to ramp distension (vehicle vs 3 μM OGM, *P* = .9135; vehicle vs 30 μM OGM, *P* = .7072; 1-way ANOVA [main effect: *P* = .6056; F(2, 12) = .5232] with Dunnett’s multiple comparison test; N = 3–6). (*Ci*) Response profile (*P* = .4615; 2-way ANOVA; N = 5–6) and (*Cii*) peak change in afferent activity in response to bradykinin (1 μM) stimulation (vehicle vs 3 μM OGM, *P* = .9945; vehicle vs 30 μM OGM, *P* = .4618; 1-way ANOVA [main effect: *P* = .4315; F(2, 14) = .8931] with Dunnett’s multiple comparison test; N = 5–6). (*Di*) Response profile (*P* = .0524; 2-way ANOVA; N = 6) and (*Dii*) peak change in afferent activity in response to capsaicin (1 μM) stimulation (vehicle vs 3 μM OGM, *P* = .3043; vehicle vs 30 μM OGM, *P* = .2195; 1-way ANOVA [main effect: *P* = .1959; F(2, 15) = 1.821] with Dunnett’s multiple comparison test; N = 6).

Comparable to studies performed in tissue from GPR68^-/-^ mice, responses to ramp distension (*P* = .6056; N = 3–6), bradykinin (*P* = .4315; N = 5–6), and capsaicin (*P* = .1959; N = 6) were similar between treatment groups (1-way ANOVA main effect) (Figure 10).

### Positive Allosteric Modulation of GPR68 Potentiates Colonic Afferent Responses to Acid and Noxious Mechanical Distension

To further explore the functional consequences of GPR68 activation, we next examined the effects of Ogerin, a selective positive allosteric modulator of GPR68,^33^ on colonic afferent responses to acidic and mechanical stimuli.

**Figure 11:**
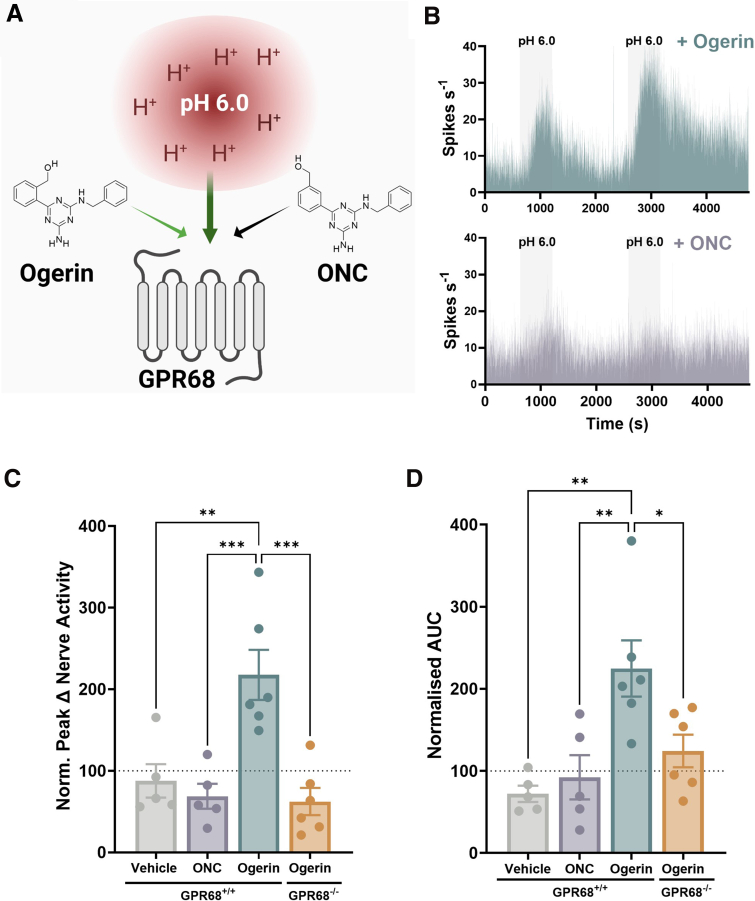
**The GPR68 PAM Ogerin potentiates colonic afferent responses to acid.** (*A*) Schematic of the experimental paradigm assessing colonic afferent responses to bath application of pH 6.0 following treatment with the GPR68 PAM, Ogerin, or its inactive analogue ONC. (*B*) Representative rate histograms of repeated afferent stimulation with pH 6.0. The second response consisted of pretreatment with either Ogerin (*top*) or ONC (*bottom*). (*C*) Peak change in afferent activity of response 2 (post-drug application) normalized to response 1 (pre-drug application) for vehicle, ONC, or Ogerin (in GPR68^+/+^ and GPR68^-/-^ mice) treated groups (Ogerin vs ONC, *P* = .0005; Ogerin vs vehicle, *P* = .0019; Ogerin vs GPR68^-/-^, *P* = .0002; 1-way ANOVA [main effect: *P* = .0002; F(3, 18) = 11.36]; with Dunnett’s multiple comparisons test; N = 5–6 animals per group). (*D*) AUC of response 2 (post-drug application) normalised to response 1 (pre-drug application) for vehicle, ONC, or Ogerin (in GPR68^+/+^ and GPR68^-/-^ mice) treated groups (Ogerin vs ONC, *P* = .0047; Ogerin vs vehicle, *P* = .0014; Ogerin vs GPR68^-/-^, *P* = .0237; 1-way ANOVA [main effect: *P* = .002; F(3, 18) = 7.367]; with Dunnett’s multiple comparisons test; N = 5–6 animals per group).

Consistent with a role for GPR68 in acid-evoked colonic afferent activation, pretreatment with Ogerin, but not the Ogerin negative control (ONC), significantly increased the peak change and AUC of colonic afferent responses to pH 6.0 Krebs (Figure 11*A–B*) compared with vehicle control; this effect was no longer observed in colonic afferent recordings made from GPR68^-/-^ mice, confirming the specificity of Ogerin for GPR68 receptor activity (Peak: Ogerin vs ONC; *P* = .0005; Ogerin vs vehicle; *P* = .0019; Ogerin vs GPR68^-/-^; *P* = .0002; 1-way ANOVA with Dunnett’s multiple comparisons test; AUC: Ogerin vs ONC; *P* = .0047; Ogerin vs vehicle; *P* = .0014; Ogerin vs GPR68^-/-^; *P* = .0237; 1-way ANOVA with Dunnett’s multiple comparisons test; N = 5–6) (Figure 11*C–D*).

**Figure 12:**
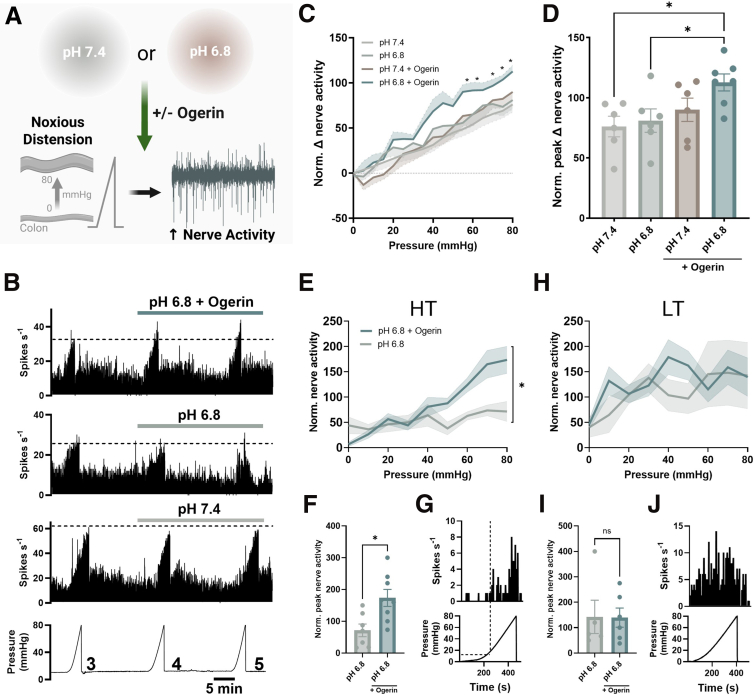
**The GPR68 PAM Ogerin potentiates colonic afferent responses to noxious mechanical distension.** (*A*) Schematic of experimental paradigm assessing the effects of the GPR68 PAM, Ogerin (applied intraluminally), on colonic afferent responses to ramp distension. (*B*) Representative rate histograms and corresponding pressure traces from recordings showing afferent responses to ramp distensions 3–5 in pH 7.4, 6.8 and pH 6.8 + Ogerin (100 μM) groups. The *dotted line* highlights the sensitization of afferent responses to distension in the pH 6.8 + Ogerin group. (*C*) Afferent response profiles of ramp distension 5 (0–80 mmHg) in pH 7.4 and pH 6.8 groups with and without Ogerin, normalized (norm.) to ramp distension 3 (∗*P* < .05; 2-way repeated measures ANOVA [main effect: *P* = .0493; F(3, 21) = 3.087] with Dunnett’s multiple comparisons test). Shaded area is SEM. (*D*) Peak change in nerve activity at 80 mmHg during Ramp 5 (post-drug application), expressed as a percentage of Ramp 3 (pre-drug application), in pH 7.4 and pH 6.8 groups with and without Ogerin (pH 7.4 vs pH 6.8 with Ogerin, *P* = .0194; pH 6.8 vs pH 6.8 with Ogerin, *P* = .0474; 1-way ANOVA [main effect: *P* = .0283; F(3, 21) = 3.681] with Dunnett’s multiple comparisons test) (N = 6–7 animals). (*E*) HT response profile of ramp distension 5 (0–80 mmHg) in pH 6.8 groups with and without Ogerin, normalized (norm.) to ramp distension 3 (*P* = .0192; 2-way ANOVA; F(1, 13) = 7.137; n = 7–8 units). (*F*) Peak change in HT unit activity at 80 mmHg during Ramp 5 (post-drug application), expressed as a percentage of Ramp 3 (pre-drug application), in pH 6.8 groups with and without Ogerin (*P* = .0106; unpaired *t*-test (t, df = 2.981, 13); n = 7–8 units). (*G*) Representative rate histogram (*top*) and corresponding pressure trace (*bottom*) from a high-threshold fiber showing afferent responses at luminal pressures exceeding 15 mmHg. (*H*) LT response profile of ramp distension 5 (0–80 mmHg) in pH 6.8 groups with and without Ogerin, normalized (norm.) to ramp distension 3 (*P* = .5472; 2-way ANOVA; F(1, 9) = .3912; n = 5–6 units). (*I*) Peak change in LT unit activity at 80 mmHg during Ramp 5 (post-drug application), expressed as a percentage of Ramp 3 (pre-drug application), in pH 6.8 groups with and without Ogerin (*P* = .9678; unpaired *t*-test (t, df = .042, 9); n = 5–6 units). (*J*) Representative rate histogram (*top*) and corresponding pressure trace (*bottom*) from a low-threshold fiber showing afferent responses from 0–80 mmHg.

To further elucidate the contribution of GPR68 to the sensitization of mechanosensitive colonic afferents, we investigated the effect of Ogerin on colonic afferent responses to luminal distension with pH 7.4 Krebs buffer and weakly acidified pH 6.8 Krebs buffer (Figure 12*A–B*). Consistent with its role as a positive allosteric modulator of GPR68, pretreatment with Ogerin (100 μM) had no significant effect on the colonic afferent firing response to ramp distension with pH 7.4 buffer (pH 7.4 vs pH 7.4 with Ogerin; *P* = .6286; 1-way ANOVA with Dunnett’s multiple comparisons test; N = 6–7) (Figure 12*C–D*). However, Ogerin significantly enhanced the afferent response to colonic distension with pH 6.8 buffer at noxious distending pressures (75 mmHg; *P* = .0162; 80 mmHg; *P* = .02; repeated measures 1-way ANOVA with Dunnet’s multiple comparisons test; N = 6–7) (Figure 12*C*), such that a significant increase in peak afferent firing during ramp distension was observed (pH 7.4 vs pH 6.8 with Ogerin; *P* = .0194; pH 6.8 vs pH 6.8 with Ogerin; *P* = .0474; N = 6–7) (Figure 12*D*). Luminal distension with pH 6.8 buffer alone produced a comparable increase in colonic afferent activity to distension with pH 7.4 buffer. Furthermore, unit classification revealed that intraluminal incubation with Ogerin significantly increased the response of high-threshold (HT), but not low-threshold (LT), units to distension at pH 6.8 (peak change: *P* = .0106; unpaired *t*-test) (Figure 12*E–J*).

## Discussion

Abdominal pain is a leading cause of morbidity in individuals with IBD, often persisting during remission, underscoring the limitation of current therapies for the resolution of pain.^34^ A critical unmet clinical need therefore exists for the development of novel treatment strategies that not only target inflammation but also nociceptive signaling. To address this, we investigated the contribution of GPR68, a proton-sensing GPCR implicated in intestinal inflammation and fibrosis,^21^^,^^23^^,^^25^ to the activation of colonic afferents and DRG sensory neurons.

Consistent with reports of a proinflammatory role for GPR68 in colitis,^22^^,^^25^ we showed that GPR68^-/-^ mice exhibited significantly less disease activity following DSS-induced acute colitis, and in silico analysis of our reported RNA sequencing (RNA-seq) data revealed increased expression of GPR68 alongside other proton-sensing GPCRs in colonic biopsies from individuals with IBD.^30^ These data support the potential utility of GPR68 antagonists for the treatment of inflammation in IBD. Furthermore, transcriptomic analysis of murine colon-projecting sensory neurons showed that GPR68 expression was markedly higher than other proton-sensing GPCRs and was particularly enriched in *Trpv1*-expressing peptidergic nociceptors.^35^ These observations highlight a unique opportunity to target GPR68 to suppress both nociceptor signaling and inflammation in colitis.

To better understand the contribution of GPR68 to sensory signaling, we examined DRG neuron responses to acidic pH in Ca^2+^-free conditions, thereby isolating intracellular Ca^2+^ release. We observed a significant reduction in the amplitude and proportion of DRG neurons responding to weakly acidic pH (pH 6.0–6.5) in preparations from GPR68^-/-^ mice or following pretreatment of DRG neurons with the GPR68 antagonist, OGM. Prior studies similarly demonstrate that acid-induced intracellular Ca^2+^ fluxes are diminished in cells expressing mutant GPR68,^36^ and that knockdown of GPR68 in airway smooth muscle cells attenuates acid-evoked Ca^2+^ mobilization.^37^^,^^38^ Our findings, showing that pharmacological inhibition of G_q_ and PLC blocks pH 6-evoked Ca^2+^ responses, are consistent with the known G_q_-coupling of GPR68 signaling to acid,^17^ which liberates Ca^2+^ from intracellular stores via IP_3_ receptors following G_q_-mediated PLC activation and formation of IP_3_ from phosphatidylinositol 4, 5-bisphosphate (PIP_2_).

The marked reduction in acid-evoked responses in DRG neurons isolated from GPR68^-/-^ mice or following pretreatment with OGM, is also in line with GPR68 being the most highly expressed proton-sensing GPCR in colon-projecting sensory neurons.^35^ The presence of a small residual population of DRG neurons exhibiting intracellular Ca^2+^ responses to acid in Ca^2+^-free conditions further suggests the involvement of other proton sensitive pathways capable of mobilizing intracellular Ca^2+^ in sensory neurons.

We observed comparable responses to capsaicin and no difference in acid-evoked responses in DRG neurons isolated from GPR68^+/+^ and GPR68^-/-^ mice when incubated in normal Ca^2+^-containing buffer. These responses mediated by external Ca^2+^ entry are most likely due to the well-established roles of proton-sensing ion channels, such as TRPV1 and acid-sensing ion channels (ASICs), in the activation of sensory neurons by acid.^27^ Furthermore, the preservation of these responses to capsaicin and extracellular Ca^2+^-mediated acid signaling in turn demonstrates that deletion of GPR68 had not significantly impaired the activation of other proton-stimulated receptors in sensory neurons.

Importantly, this pattern of impaired acid responsiveness translated to colonic afferents, which showed markedly reduced activation at pH 5.5 in the presence of the GPR68 antagonist OGM, and virtually no response to acid in tissue from GPR68^-/-^ mice across a pH range of 4.0 to 6.5. These findings reveal a previously unrecognized role for GPR68 in mediating acid-induced activation of sensory nerve endings in the colon, resonant with ex vivo recordings of esophageal afferents.^39^ Interestingly, this included proton concentrations (pH 4.0–6.0) sufficient to activate other known acid-sensing receptors known to contribute to acid-evoked stimulation of colonic afferents, such as TRPV1. Given that responses to capsaicin were unchanged in both colonic afferents and DRG neurons from GPR68^-/-^ mice, the near-complete loss of acid-evoked afferent signaling strongly suggests that GPR68 is essential for enabling or amplifying sensory neuron activation in response to acid, potentially through facilitation of other proton-sensitive pathways like TRPV1.

Further evidence supporting the role of GPR68 in acid-mediated activation of colonic nociceptors was provided by experiments using Ogerin, a positive allosteric modulator (PAM) that is currently used as a research tool compound and has been shown to be bioavailable and brain-penetrant in mice, consistent with its reported effects on hippocampal plasticity and behavior.^40^^,^^41^ Ogerin selectively shifts GPR68’s pH response curve^33^ and enhances GPR68 signaling in various tissues.^42^, ^43^, ^44^ In line with this mechanism, Ogerin potentiated colonic afferent responses to acid at pH 6.0 and sensitized the colonic afferent response to luminal distension with weakly acidic (pH 6.8), but not neutral (pH 7.4), Krebs buffer. Sensitization by Ogerin was observed only at noxious distension pressures, consistent with the selective expression of GPR68 in colonic nociceptors and was further supported by spike-sorted afferent recordings showing enhanced responses of HT units (>15 mmHg). Interestingly, HT unit activity appeared elevated at lower distension pressures following pH 6.8 application. Although this effect was not apparent in the presence of Ogerin, no significant differences were detected between groups at low pressures, and further investigation would be needed to clarify this observation.

Despite modulating the activity of distension-sensitive fibers, and in contrast to recent reports suggesting that GPR68 can be activated by membrane stretch and shear stress,^45^^,^^46^ consistent with the presence of a conserved Helix 8 structural motif shared among mechanosensitive GPCRs,^47^ we found no difference in the magnitude of the colonic afferent response to colorectal distension between wild-type and GPR68^-/-^ mice. Although this finding does not exclude the possibility that GPR68 is mechanosensitive, it suggests a likely functional redundancy in its role in mechanotransduction within colonic afferents, especially when compared with the established contributions of other mechanosensitive channels such as TRPA1 and PIEZO2, whose loss results in a marked reduction in colonic afferent responses to distension.^48^^,^^49^ Nevertheless, our data generated using Ogerin demonstrate that, although GPR68 may not function as a primary mechanotransducer in colonic afferents, its activation can still enhance LSN sensitivity to mechanical stimuli.

In summary, our findings reveal a critical role of GPR68 in the activation of colonic afferents by acid and demonstrate its capacity to sensitize colonic afferent responses to noxious distension of the bowel, as shown through both pharmacological and genetic approaches. These results position GPR68 as a key contributor to colonic nociception during tissue acidosis and support the need for further investigation into its role in pain signaling in GI disorders where acidosis is a prominent feature, such as colitis. Importantly, GPR68 antagonists are currently in clinical development,^50^ highlighting the translational potential of our findings and the therapeutic relevance of targeting this receptor.

## Methods

### Ethical Approval

All experimental procedures performed on animals were conducted in compliance with the Animals (Scientific Procedures) Act 1986 Amendment Regulations 2012 under Project License PP5814995 granted to Ewan St. John Smith by the United Kingdom Home Office with approval from the University of Cambridge Animal Welfare Ethical Review Body. In addition, animals were euthanized by rising concentration of 100% CO_2_ followed by exsanguination in accordance with Schedule 1 of the Animals (Scientific Procedures) Act 1986 Amendment Regulations 2012.

### Animals

Adult C57BL/6J mice (8–16 weeks) were obtained from Charles River (RRID: IMSR_JAX: 000664). GPR68 knockout (GPR68^-/-^) mice (MGI ID: 5776516) were kindly gifted by Martin Hausmann and Gerhard Rogler^24^ and bred in Cambridge with C57BL/6J mice to generate wild-type (GPR68^+/+^) and heterozygous (GPR68^+/-^) littermates. Mice were housed in temperature-controlled rooms (21°C) on a 12-hour light/dark cycle with ad libitum access to food and water, nesting material, and environmental enrichment consisting of tunnels and shelters. No sex differences were observed in afferent responses to acidic pH between GPR68^+/+^ and GPR68^-/-^; therefore, data from male and female mice were pooled for all subsequent analyses.

### Reagents

Stock concentrations of Ogerin (100 mM in dimethyl sulfoxide [DMSO]) and ONC (a structurally similar inactive analogue of Ogerin; 65 mM in DMSO) were obtained from Tocris Bioscience and Sigma-Aldrich, respectively. OGM (10 mM in DMSO) was synthesized at AstraZeneca. Capsaicin (1 mM in 100% ethanol), atropine (100 mM in 100% ethanol), and nifedipine (100 mM in DMSO) were obtained from Sigma-Aldrich and bradykinin (1 mM in DMSO) was obtained from Tocris. Edelfosine (1 mM in DMSO) and YM-254890 (0.1 mM in DMSO) were obtained from Cambridge Biosciences and CliniSciences, respectively. Immediately before use, compounds were diluted to their final working concentrations in either extracellular solution (ECS) or Krebs buffer, as appropriate. 1M HCl was used to adjust the pH of ECS or Krebs buffer.

### In Silico Analysis of Human Colonic Biopsy Bulk RNA-seq and Murine Colonic Sensory Neuron Single-cell RNA-seq Datasets

Bulk RNA sequencing data from colonic biopsies were obtained from a previously published dataset from Higham et al,^30^ consisting of samples from patients with UC (n = 9 biopsies from N = 9 patients), CDN (n = 8 biopsies from N = 7 patients), and CDT (n = 11 biopsies from N = 7). All patients with IBD had reported abdominal pain within the 4 weeks prior to endoscopy.

Samples had also been collected from the colon of people with abdominal pain, but no visible inflammation, diagnosed as recurrent abdominal pain (RAP; n = 21 biopsies from N = 16) and NI (n = 14 biopsies from N = 8) obtained from individuals with no symptoms of abdominal pain or evidence of mucosal inflammation on endoscopy.

Single-cell RNA sequencing (scRNA-seq) data from murine DRG neurons were acquired from Hockley et al.^35^ In brief, this study performed transcriptomic profiling and unsupervised clustering of 314 retrogradely traced mouse colonic sensory neurons isolated from thoracolumbar (TL) and lumbosacral (LS) DRG.^35^

### DSS-induced Acute Colitis

Following baseline weight measurements on day 0, GPR68^+/+^ and GPR68^-/-^ mice of either sex received drinking water supplemented with 1.5% DSS (Thermo Fisher Scientific) for 5 consecutive days, followed by normal drinking water for an additional 2 days. Control mice were provided with normal drinking water without DSS throughout the experiment. Mice were monitored daily, and disease severity was quantified using a disease activity index (DAI) as conducted previously.^31^ The DAI was calculated as the sum of scores for weight loss (0 = none; 1 = 1%–5%; 2 = 5%–10%; 3 = 10%–15%; 4 = >15%), stool consistency (0 = normal; 2 = loose stool; 4 = watery diarrhea), and presence of blood in stool (0 = none; 2 = slight bleeding; 4 = gross bleeding).

At the end of the study, mice were euthanized as described above, and colon length and spleen weight were measured.

### Colon Histology

A segment of distal colon (∼1 cm from the rectum) was dissected and fixed overnight at 4°C in 4% (w/v) formaldehyde (Sigma Aldrich). The following day, the tissue was washed in phosphate buffered saline (PBS) and cryoprotected overnight at 4°C in 30% (w/v) sucrose (Thermo Fisher Scientific). Tissue was then embedded in Shandon M-1 Embedding Matrix (Thermo Fisher Scientific), frozen on dry ice, and stored at −80°C. Serial 20-μm sections were made using a Leica CM3000 cryostat and stored at −20°C until staining. Sections were stained with hematoxylin (0.2% w/v; Sigma Alderich; nuclei stain), and eosin (0.5% w/v; Acros Organics; cytoplasmic counterstain), as described previously,^51^ and mounted with glycerol. A NanoZoomer S360 and NDP.view2 (Hamamatsu) was used to image and visualize the staining, respectively.

Histopathological scoring was conducted by blinded investigators and evaluated 3 parameters: inflammatory cell infiltration (0 = none; 1 = increased immune nuclei in lamina propria; 2 = extension into submucosa; 3 = transmural infiltration), crypt damage (0 = intact crypts; 1–5 = increasing severity from partial crypt loss to confluent epithelial erosion), and ulceration (0 = none; 1–5 = increasing number and extent of ulcerative foci). At least 2 sections per animal, spaced 150 μm apart, were independently scored by 2 experimenters, and the average score per mouse was analyzed.

### Isolation and Culture of Primary Mouse DRG Neurons

Mice were euthanised as described above, and thoracolumbar (T12-L5) DRG were dissected, with spinal levels selected due to their innervation of the distal colon.^52^ Isolated DRG were transferred to 2 mL ice-cold Leibovitz L-15 medium supplemented with GlutaMAX and 2.6% (v/v) NaHCO_3_. Tissues were enzymatically digested at 37°C using type 1A collagenase (1 mg/mL, 15 min) followed by trypsin (1 mg/mL, 30 min), both prepared with 6 mg/mL bovine serum albumin (BSA). Following digestion, DRG were resuspended in 2 mL of dissociation medium (Leibovitz L-15 with GlutaMAX, 2.6% [v/v] NaHCO_3_, 10% fetal bovine serum [FBS], 1.5% [v/v] glucose, and 300 U/mL penicillin/0.3 mg/mL streptomycin). Mechanical trituration was performed with increasing force, and the resulting suspension was centrifuged at 100 g to collect the supernatant across 5 sequential triturations.

The pooled supernatants were centrifuged again (5 minutes, 100 g), pellets resuspended, and 50 μL of cell suspension was plated onto laminin-coated poly-D-lysine-coated glass-bottom dishes (MatTek). Cultures were incubated at 37°C for 2 to 3 hours to allow cell attachment, after which dishes were flooded with additional dissociation medium and maintained overnight at 37°C in 5% CO_2_.

### Ca^2+^ Imaging

Cultured DRG neurons were incubated with 10 μM Fluo-4-AM (Invitrogen), diluted in ECS containing (in mM): 140 NaCl, 4 KCl, 1 MgCl_2_, 2 CaCl_2_, 4 D-glucose, and 10 HEPES, adjusted to pH 7.4 with NaOH and 290-310 mOsm with sucrose. Incubation was carried out for 30 to 45 minutes at room temperature protected from light, following which cells were gently washed with ECS, and the glass-bottom dishes were mounted onto the stage of an inverted Nikon Eclipse TE-2000S microscope for imaging.

Fluo-4 was excited with a 470 nm LED light source (Cairn Research), and emission detected at 520 nm using Micro-manager software (v1.4; NIH). Intracellular Ca^2+^ transients were recorded using a Retiga Electro CCD camera (Photometrics), and images were captured at 2.5 frames per second with 100 ms exposure time. A gravity-fed perfusion system (Warner Instruments) with 250 μm perfusion tip (Digitimer) was used to continuously superfuse cells with ECS or test solutions at a rate of ∼0.5 mL/min.

### Ca^2+^ Imaging Protocols

To assess drug responses, cells were superfused with ECS for 10 seconds to establish baseline fluorescence, and for 4 minutes between treatments to allow for recovery. Acidic pH was applied to cells for 10 to 30 seconds followed by 10 seconds capsaicin (1 μM). The ECS pH was adjusted immediately preceding the experiment using HCl (1 M). For antagonist studies, cells were preincubated for 10 minutes with 200 μL OGM (300 nM) prior to imaging, and the inhibitor present throughout recordings. At the end of each protocol, a 10-second application of 50 mM KCl was used to depolarize neurons, confirm viability, and enable fluorescence normalization. For studies where extracellular Ca^2+^ was omitted, cells were incubated with Ca^2+^-free ECS for 10 minutes following Fluo-4 incubation. Ca^2+^ free ECS contained (in mM): 140 NaCl, 4 KCl, 2 MgCl_2_, 4 glucose, 10 HEPES, and was supplemented with 1 EGTA to chelate residual Ca^2+^. To equilibrate intracellular Ca^2+^ prior to 50 mM KCl application, cells were perfused with normal ECS for 10 minutes following the application of acidic pH in Ca^2+^-free ECS.

### Ca^2+^ Imaging Data Analysis

Image analysis was conducted using Fiji/ImageJ (NIH). Regions of interest (ROIs) were manually drawn around individual neurons, and mean pixel intensity (F) per frame was computed. Using custom-written scripts in RStudio, background fluorescence was subtracted, and intensity values were normalized to the maximal fluorescence elicited by 50 mM KCl (F_pos_). Neurons were considered responders if their F/F_pos_ increased by ≥0.1 during stimulation. For each condition, both the proportion of responsive neurons and the average response magnitude were compared across groups.

### Ex Vivo Colonic Afferent Recording

Mice were euthanized as described above, and the distal colon along with the associated LSN was isolated via laparotomy. Tissue was immediately transferred to a Sylgard-coated recording chamber maintained at 32 to 35°C. The colorectum was cannulated at both ends and perfused luminally (100 μL/min) and serosally (7 mL/min) with carbogenated Krebs buffer (95% O_2_, 5% CO_2_). The Krebs buffer consisted of (in mM): 124 NaCl, 4.8 KCl, 1.3 NaH_2_PO_4_, 25 NaHCO_3_, 1.2 MgSO_4_, 11.1 D-glucose, and 2.5 CaCl_2_, and was supplemented with nifedipine and atropine (both at 10 μM) to suppress smooth muscle activity.

The LSN was dissected free from surrounding connective tissue and fat, and multiunit afferent activity was recorded using a borosilicate glass suction electrode, as conducted previously.^53^, ^54^, ^55^ Electrical signals were amplified (gain 5 kHz), bandpass filtered (100–1300 Hz; Neurolog, Digitimer Ltd) and digitized (20 kHz; micro1401, CED). Signals were visualized in real time using Spike2 software (v8.23; CED), with digital filtering for 50 Hz noise (Humbug, Quest Scientific).

### Electrophysiology Protocols

Colonic afferent preparations were left for 30 to 40 minutes to stabilize prior to protocol initiation. Krebs buffer was adjusted to the desired pH with HCl (1 M) immediately before application. To assess the contribution of GPR68 to colonic afferent responses to extracellular acidification (across the full range of acidic stimuli), preparations from GPR68^+/+^ and GPR68^-/-^ mice were exposed to Krebs buffer at decreasing pH values (pH 6.5 to 4.0) via serosal perfusion to generate pH-response curves. A recovery period of 15 to 20 minutes was allowed between each stimulation to prevent desensitization.

To further examine the role of GPR68, colonic afferent preparations were exposed to acidic Krebs buffer (pH 6.0 or 5.5) in the presence of either the GPR68-positive allosteric modulator, Ogerin (100 μM), or the selective antagonist OGM (3 μM, 30 μM). Protocols consisted of 3 repeated pH challenges at 20-minute intervals, with Ogerin or OGM included only during the second stimulation to assess their modulatory effects. In GPR68^-/-^ or OGM-treated groups, neuronal functionality was confirmed at the end of the protocol by applying bradykinin (1 μM), capsaicin (1 μM), and a ramp distension (0–80 mmHg).

To assess the role of GPR68 in mechanical hypersensitivity, wild-type colonic afferent preparations were exposed to a series of 5 ramp distensions at 15-minute intervals. Luminal pressure was increased to 80 mmHg (pressure transducer: Neurolog model NL108) over ∼250 seconds, which is above the threshold for visceral mechanoreceptors.^56^^,^^57^ Krebs buffer at physiological or slightly acidic pH (pH 7.4 or 6.8, respectively) in the presence or absence of Ogerin (100 μM) was perfused intraluminally (Harvard Apparatus, Pump 11 Elite) between ramps 3 and 5; pH 6.8 was used to minimize direct activation of colonic afferents, which could confound assessment of mechanosensitivity.

### Electrophysiology Data Analysis

Electrophysiological activity of the LSN was analysed using Spike2 software (v8.23; CED). Multi-unit spike detection was performed by applying a threshold set at twice the background noise level (30–50 μV). For protocols involving direct application of acidic Krebs buffer, spike counts were binned to calculate average firing frequency in 60-second intervals. The average firing frequency during the 6 minutes prior to acid application served as the baseline to which the peak firing rate during acid exposure was normalized. Peak changes in firing rate and AUC were calculated for each pH challenge and used to compare responses across genotypes and treatment conditions. For Ogerin and OGM datasets, the second response (in the presence of the modulators) was compared with the first response (pre-modulator incubation).

For ramp distension protocols, changes in firing rate were quantified relative to baseline firing (3 minutes prior to distension). Nerve activity was assessed at 5-mmHg increments, and from these data, both the peak firing rate (at 80 mmHg) and AUC were calculated. The response to ramp 5 (post-drug) is expressed as a percentage of the response to ramp 3 (pre-drug). A post-treatment response of ∼100% indicates no change in distension-evoked firing. Single-unit activity was discriminated using the spike sorting function in Spike2 as described previously.^58^ Units were classified as LT or HT, with HT units defined by responses to noxious distension pressures >15 mmHg, and LT units defined by responses at pressures <15 mmHg that reached a plateau rapidly. For analysis, data are presented as nerve activity normalized to the firing rate achieved at 80 mmHg during Ramp 3 (uncorrected to baseline).

### Statistics

Normality was assessed, and corresponding parametric or nonparametric statistical tests were conducted. Data are presented as mean ± standard error of the mean (SEM), and statistical significance was defined as *P* ≤ .05. In figures, asterisks denote levels of significance as follows: ∗*P* < .05; ∗∗*P* < .01; ∗∗∗*P* < .001; ∗∗∗∗*P* < .0001; ns, not significant. For Ca^2+^ imaging experiments, ‘n’ refers to the number of individual culture dishes analyzed, whereas ‘N’ denotes the total number of animals from which these cultures were derived. In electrophysiological recordings, N represents the number of animals used. All statistical analyses were conducted using GraphPad Prism (version 10.4.2; GraphPad Software). Schematics are included throughout the manuscript to aid interpretation of experimental design (created with BioRender.com and ChemDraw [v21.1.2]).
